# Variations in the Anatomy of the Third Common Digital Nerve and Landmarks to Avoid Injury to the Third Common Digital Nerve With Carpal Tunnel Release

**Published:** 2008-11-03

**Authors:** Nitin J. Engineer, Ron Hazani, Arian Mowlavi, Michael W. Neumeister, W. P. Andrew Lee, Bradon J. Wilhelmi

**Affiliations:** Division of Plastic and Reconstructive Surgery, School of Medicine, University of Louisville, Louisville, KY

## Abstract

**Background:** The third common digital nerve (TCDN) has been described as the most commonly injured digital nerve during carpal tunnel release (CTR). Anatomic variations of the origin and course of the TCDN from the median nerve may place this structure at risk. Anatomic landmarks may be useful to predict the location of the TCDN to minimize the risk for injury to this structure during CTR. **Methods:** Twenty cadaveric hands were used to determine the origin and course of the TCDN. The origin of the TCDN from the median nerve was identified in relation to the transverse carpal ligament (TCL), cardinal line, and superficial palmar arch. The course of the TCDN was inspected in relation to the scaphoid tubercle and ring finger. **Results:** Three specific anatomic variations for the origin of the TCDN were identified: type 1 originating proximal to the distal edge of the TCL (3 of 20 patients), type 2 originating distal to the TCL but proximal to the superficial palmar arch (14 of 20 patients), and type 3 originating distal to the TCL and at or distal to the superficial palmar arch (3 of 20 patients). The origin of the TCDN was measured as an average of 5.0 ± 1.2 mm distal to the cardinal line. The TCDN coursed along an oblique vector from the scaphoid tubercle to the midpoint of the palmar digital crease of the ring finger for type 2 or type 3 variations. Near the cardinal line, the oblique course of the TCDN traverses the vector of thelongitudinal incision used for CTR. **Conclusion:** The TCDN is one of the most frequently damaged neurological structures during CTR. Iatrogenic injury to this structure can be disabling and even devastating to patients. A detailed knowledge of the carpal tunnel and its underlying structures can prevent inadvertent injury to the TCDN. Anatomic landmarks to predict the origin and the course of the TCDN allow the surgeon to preoperatively predict the possible locations and paths of this important structure. This information can prove to be useful in avoiding injury to the TCDN by clinicians performing CTR in their practice, whether via the open or via endoscopic technique.

Carpal tunnel release (CTR) is a time-honored procedure for the treatment of carpal tunnel syndrome with uniformly excellent results and a relatively low risk of complications.^1^ However, potentially devastating adverse outcomes do occur with both the open and endoscopic techniques of CTR.^2^–^6^ Palmer and Toivonen^7^ surveyed 708 hand surgeries and it reported 450 surgically treated complications of endoscopic and open CTR over a 5-year period, which included 121 vessel lacerations, 100 median nerve lacerations, 88 ulnar nerve lacerations, 77 digital nerve lacerations, and 69 tendon lacerations. Among these outcomes, digital nerve injuries were relatively common, and more specifically, the common digital nerve of the third web space was the most frequently injured digital nerve.

Numerous anatomic studies of the median nerve in the distal forearm and hand have been conducted.^8^–^15^ However, neither of these studies have particularly examined nor documented the branching pattern of the TCDN nor has its relationship to superficial anatomic landmarks been elucidated. The *purpose* of this study is to classify this branching pattern and determine anatomic landmarks to predict the course of the TCDN in the hand. Preoperative knowledge of the site of origin and trajectory of the TCDN using superficial landmarks can minimize injury to this structure during open or endoscopic CTR.

## METHODS

**Figure 1:**
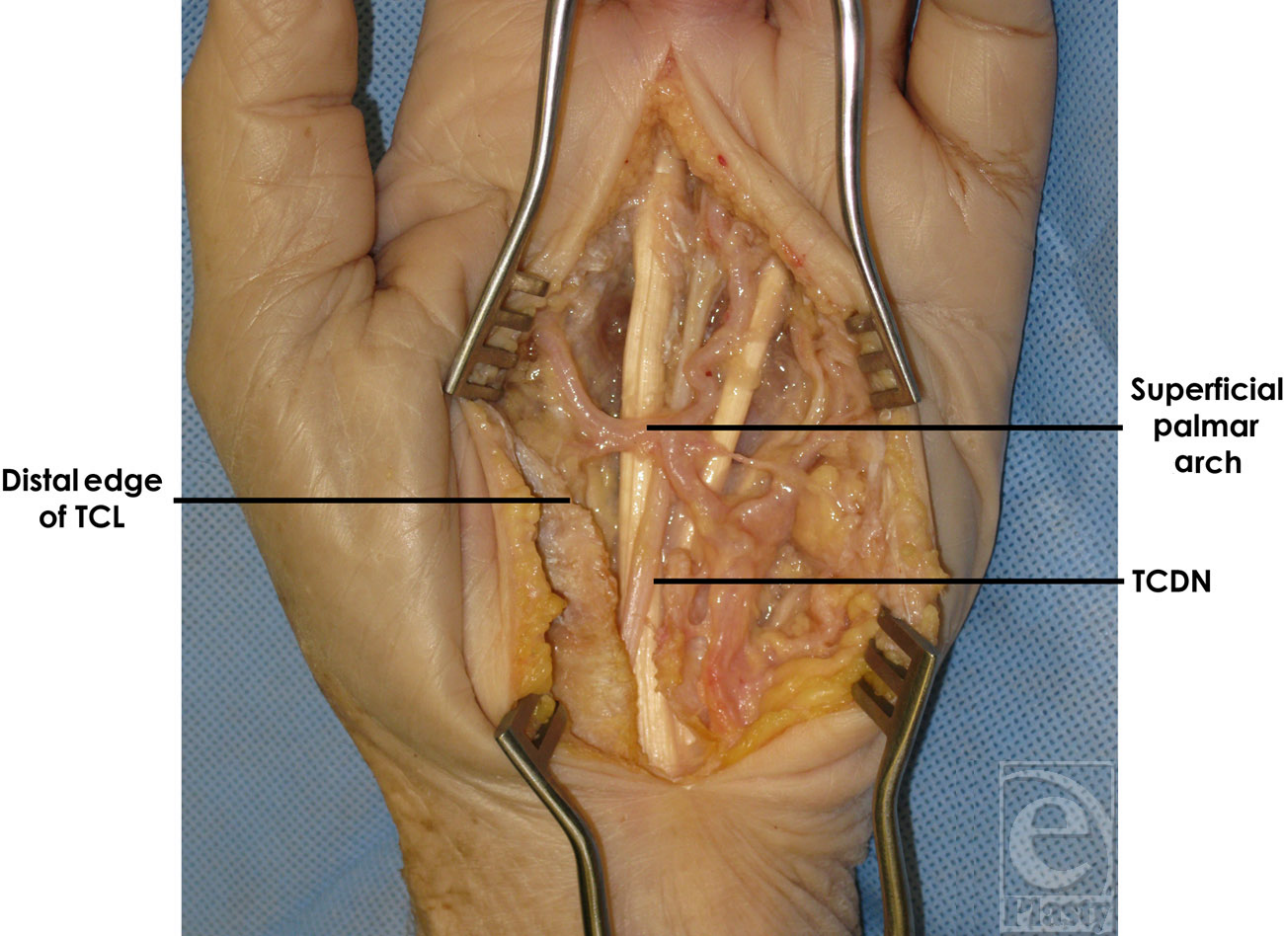
Approach to open carpal tunnel release. A longitudinal incision was made over the carpal tunnel and extended distally to expose the transverse carpal ligament (TCL) and the structures beyond it, including the third common digital nerve (TCDN) and superficial palmar arch.

Our study design involved the dissection of 20 cadaveric hands under 4.0 loupe magnification. A longitudinal incision was made over the carpal tunnel and extended distally to expose the transverse carpal ligament (TCL) and the structures beyond it, including the TCDN and superficial palmar arch (Fig 1). As performed during a standard open CTR procedure, the TCL was divided. We identified and dissected the contents of the carpal tunnel and separated them from each other and surrounding structures. The median nerve and its distal extensions, including the TCDN, were skeletonized and the origin of the TCDN was inspected in relation to the distal edge of the TCL.

**Figure 2:**
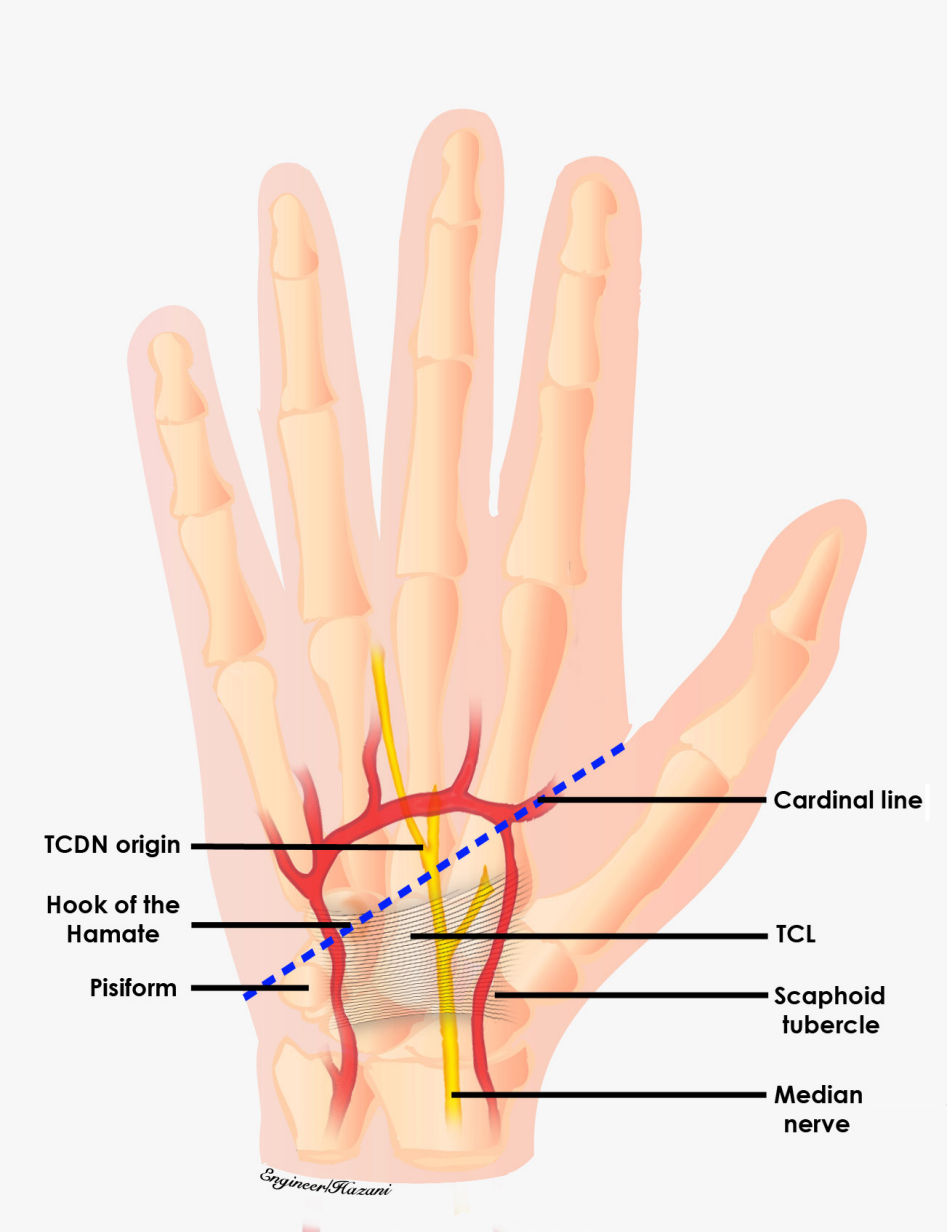
The origin of the third common digital nerve (TCDN) was inspected in relation to the distal edge of the transverse carpal ligament (TCL) and measured from the cardinal line, which was used as an external landmark.

**Figure 4:**
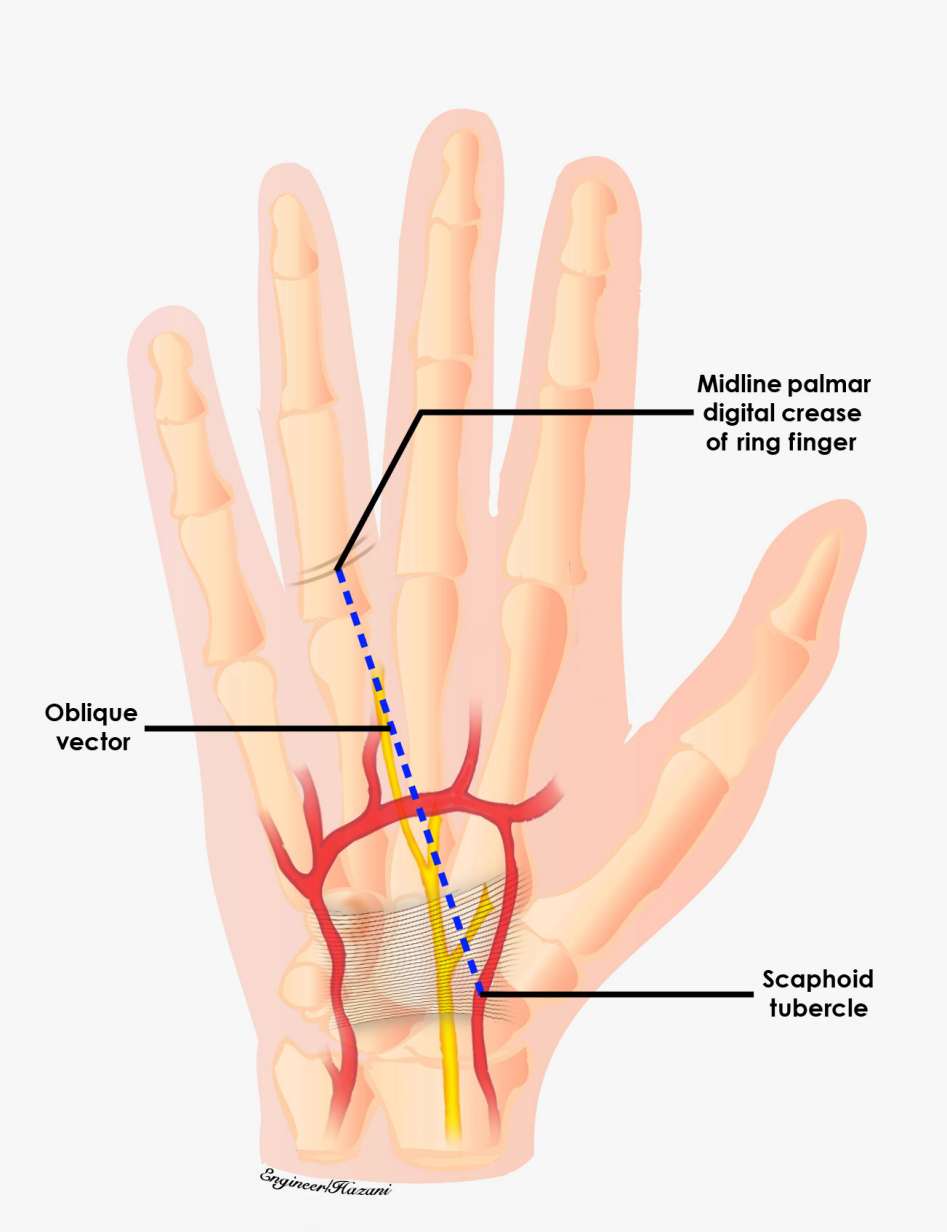
As the third common digital nerve coursed distally, its trajectory was compared with another external landmark consisting of an oblique vector from the scaphoid tubercle to the midline of the ring finger palmar digital crease.

This origin was further measured from Kaplan's cardinal line, which was used as an external landmark (Fig 2). We followed Hurst's description of this line, which extends from the apex of the interdigital fold between the thumb and index finger, a point that remains consistent despite abduction or adduction of the thumb, to the hook of the hamate, which can be consistently palpated noninvasively.^16^,^17^ Whether the TCDN originated proximally or distally to the superficial palmar arch, was also documented (Figs 3a–3c). As the TCDN coursed distally, its trajectory was compared with another external landmark consisting of an oblique vector from the scaphoid tubercle to the midline of the ring finger palmar digital crease (Fig 4).

## RESULTS

Three anatomic variations of the TCDN were identified relative to the TCL and superficial palmar arch. In 3 of 20 dissections, the TCDN originated from the median nerve within the carpal tunnel proximal to the distal edge of the TCL. This variation was labeled as type 1 (Fig 5a). In 14 of 20 dissections, the TCDN origin was found distal to the TCL but proximal to the superficial palmar arch. This was designated as type 2 (Fig 5b). Finally, 3 of 20 dissections revealed the TCDN to originate at or distal to the superficial palmar arch and were labeled as type 3 (Fig 5c). On an average, the origin of the TCDN was measured at 5.0 ± 1.2 mm distal to the cardinal line (Figs 6a and 6b).

**Figure 7:**
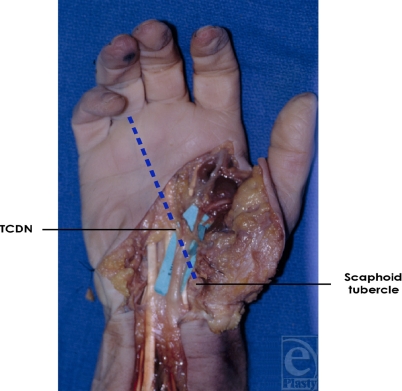
Third common digital nerve (TCDN) trajectory. The TCDN was found to reliably course along an oblique vector from the scaphoid tubercle to the midpoint of the palmar digital crease of the ring finger for type 2 and type 3 variations.

**Figure 8:**
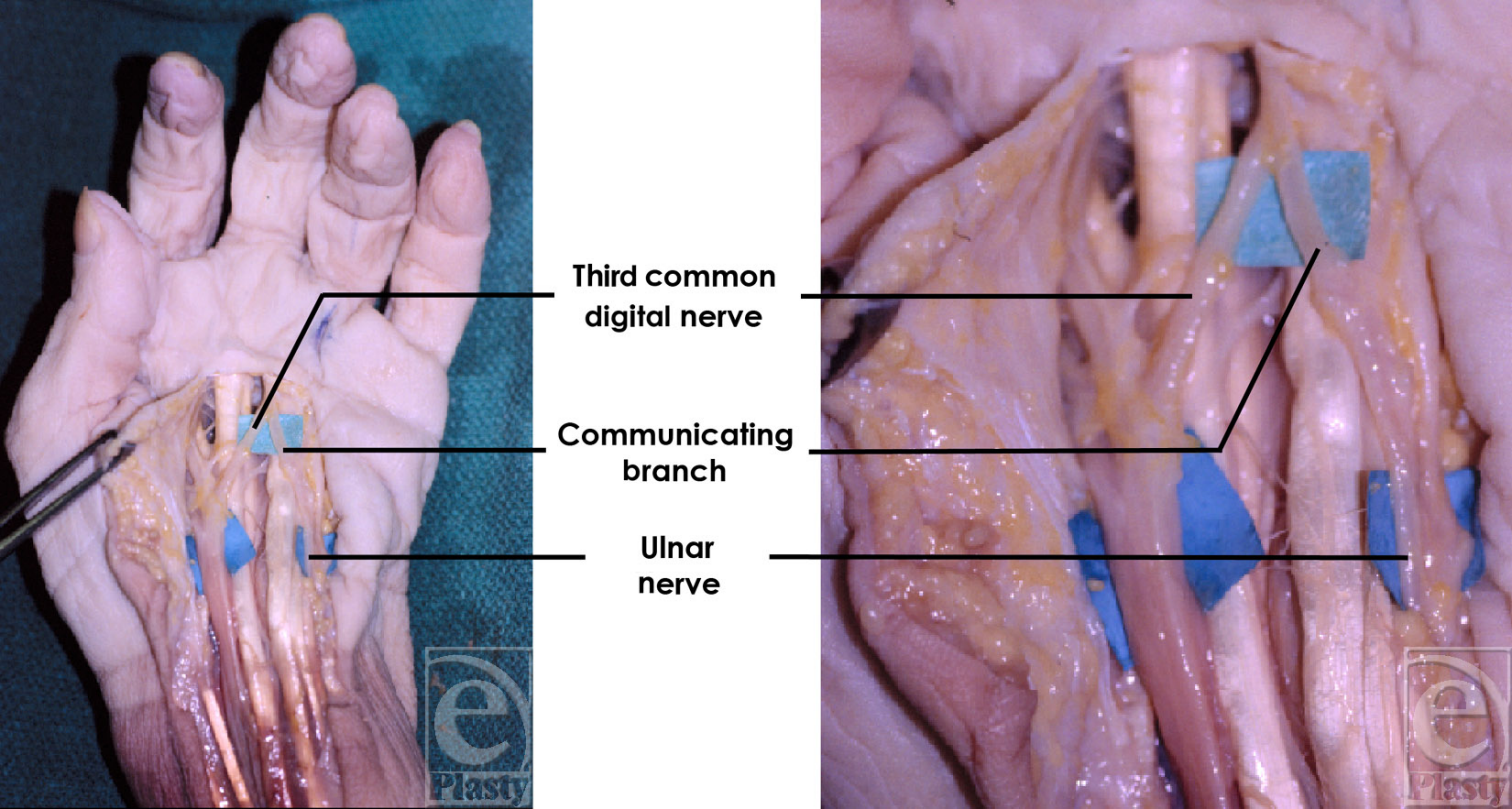
Ten of 20 third common digital nerve dissections exhibited a communicating branch to the ulnar nerve.

The natural course and trajectory of the TCDN was also examined during our anatomic dissections. It was found to reliably course along an oblique vector from the scaphoid tubercle to the midpoint of the palmar digital crease of the ring finger, for type 2 and type 3 variations (Fig 7). Furthermore, similar to prior reports, 10 of 20 TCDN dissections exhibited a communicating branch to the ulnar nerve^18^ (Fig 8).

## DISCUSSION

*Carpal tunnel syndrome* is a term given to a group of symptoms resulting from compression of the median nerve at the wrist under the TCL. These symptoms are alleviated by releasing the carpal tunnel, which can be performed via open or endoscopic technique.^19^–^23^ Several potential complications have been described related to this procedure using either technique.^2^–^6^ Among these adverse outcomes, injury to the digital nerves in the hand is of significant consequence and relatively common. In particular, the TCDN is the most frequently reported digital nerve that is injured.^7^

Though the TCDN is susceptible to a high frequency of injury in either technique of CTR, its branching pattern has not been specifically examined and documented.^24^ We were able to identify the origin of the TCDN and classify its branching pattern in 20 cadaveric hands, and these were labeled as types 1 to 3. In addition, our dissections revealed that the TCDN consistently followed an externally identifiable oblique trajectory from the scaphoid tubercle to the midpoint of the palmar digital crease.

Knowledge of the various branching patterns and external landmarks of the TCDN is pertinent to prevent unwanted injury to this structure while sharply transecting the TCL in a longitudinal fashion during CTR. The TCDN is at risk during this portion of the procedure because of its intimate relationship to the TCL. The type 1 branching pattern of the TCDN is especially susceptible to inadvertent injury, for it originates from the median nerve within the carpal tunnel. In addition, the oblique course of all variations of the TCDN puts it at great risk for injury because it crosses the longitudinal vector of the incision made to divide the TCL. If the surgeon fails to suspect its possible presence and visualize it at this potential site of injury, the TCDN may be inadvertently partially or completely divided during release of the TCL.

An interesting point of discussion is the relationship of the 3 variations of the TCDN to the 3 common approaches of CTR—open, Agee endoscopic, and Chow endoscopic techniques. In the open-CTR technique, the surgeon may equally risk injury to all 3 TCDN variations, for the longitudinal incision traverses the potential site of these origins. However, the benefit of the open technique is direct visualization of the median nerve and its branches, which may allow prevention of injury to the TCDN despite the type of origin.

With the Agee endoscopic CTR technique, we hypothesize that a single proximal incision could avoid injury to all 3 types of TCDN origins. However, if direct visualization of the median nerve and its branches using this technique is limited, the unaware surgeon could partially or completely transect the TCDN. Types 1 and 2 of the TCDN origin are at particular risk of injury in the Agee technique because they lie under or just beyond the TCL where the blade of the Agee endoscope is engaged and used to divide the TCL.

Finally, a second distal palmar incision performed in the Chow endoscopic tunnel release technique, is placed in close proximity to the intersection of a type 3 TCDN origin and the superficial palmar arch. This anatomical relationship allows for potential injury to a type 3 origin in addition to the other 2 types of TCDN origins. With all 3 techniques of CTR, the TCDN is at risk of injury. This fact further emphasizes the importance of direct visualization of the TCDN and the TCL fibers in the open and endoscopic techniques, respectively.

## CONCLUSION

Carpal tunnel release is a time-honored procedure, which requires a detailed knowledge of the carpal tunnel and its underlying structures. Although rarely encountered, the TCDN is one of the most frequently damaged neurological structures during a CTR. This iatrogenic injury can be disabling and even devastating to patients. Our study identifies clear landmarks of 3 different origins of the TCDN and its anatomic trajectory, thus allowing the surgeon to preoperatively predict the possible locations and path of this important structure. We intend for this information to be useful to help avoid injury to the TCDN by clinicians performing CTR, whether via the open or endoscopic technique.

## Figures and Tables

**Figure 3a:**
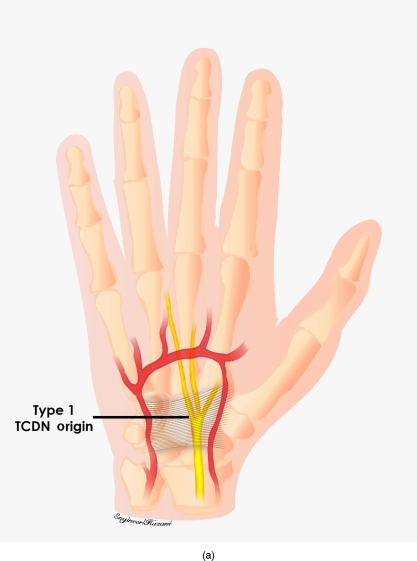
Variations of third common digital nerve (TCDN, origin). Type 1 originated from the median nerve within the carpal tunnel proximal and to the distal edge of the transverse carpal ligament (TCL).

**Figure 3b:**
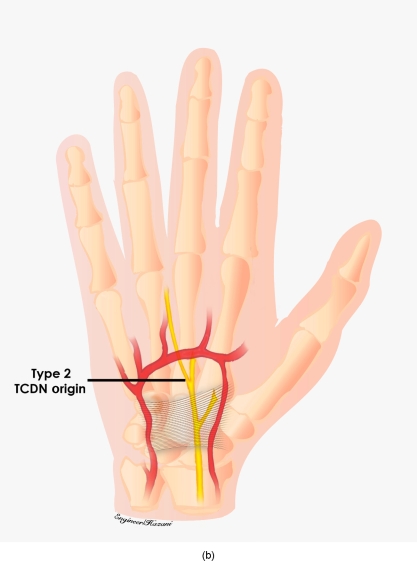
Type 2 was found distal to the TCL but proximal to the superficial palmar arch.

**Figure 3c:**
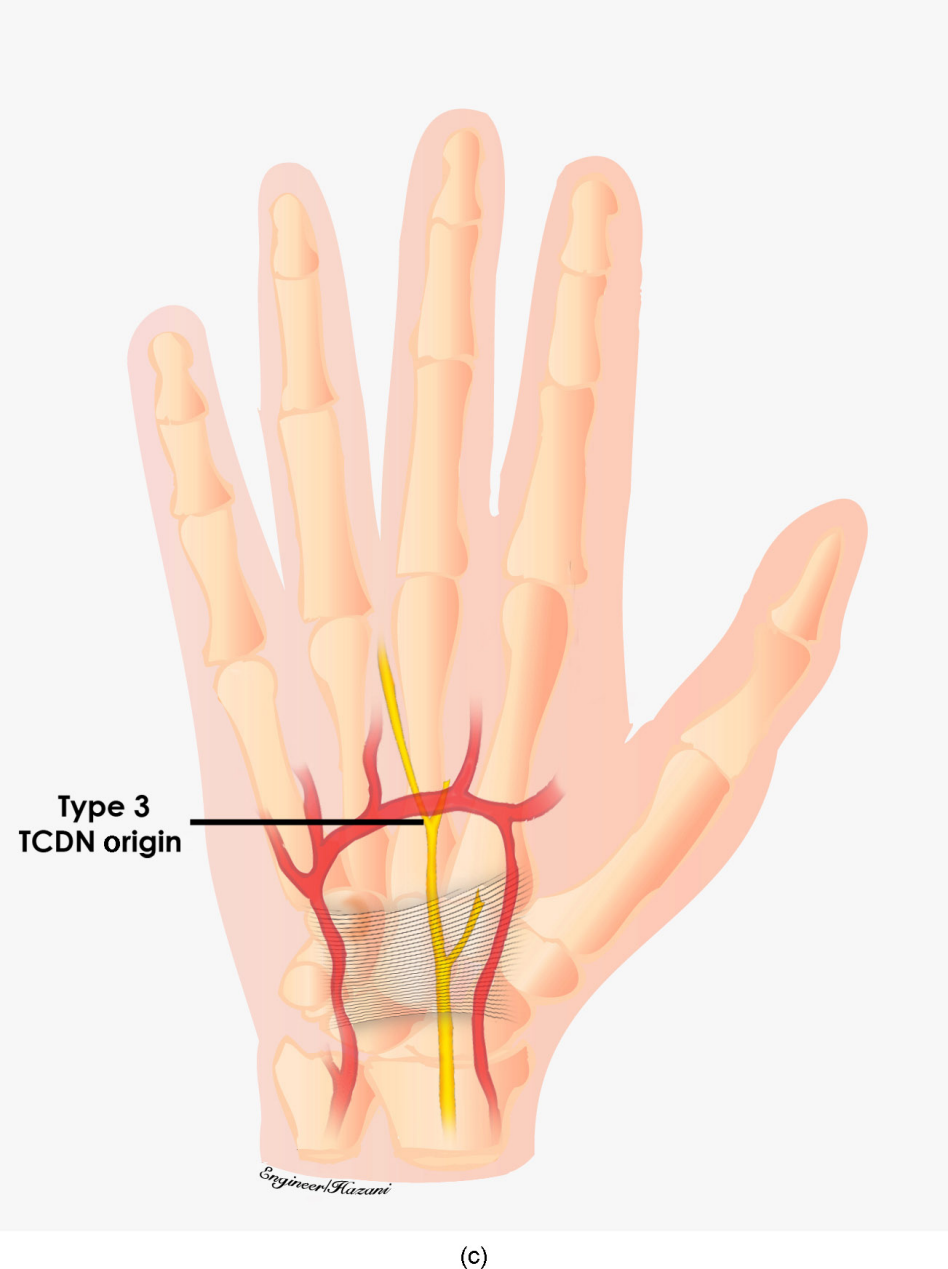
Type 3 originated distal to the TCL and at or distal to the superficial palmar arch.

**Figure 5a:**
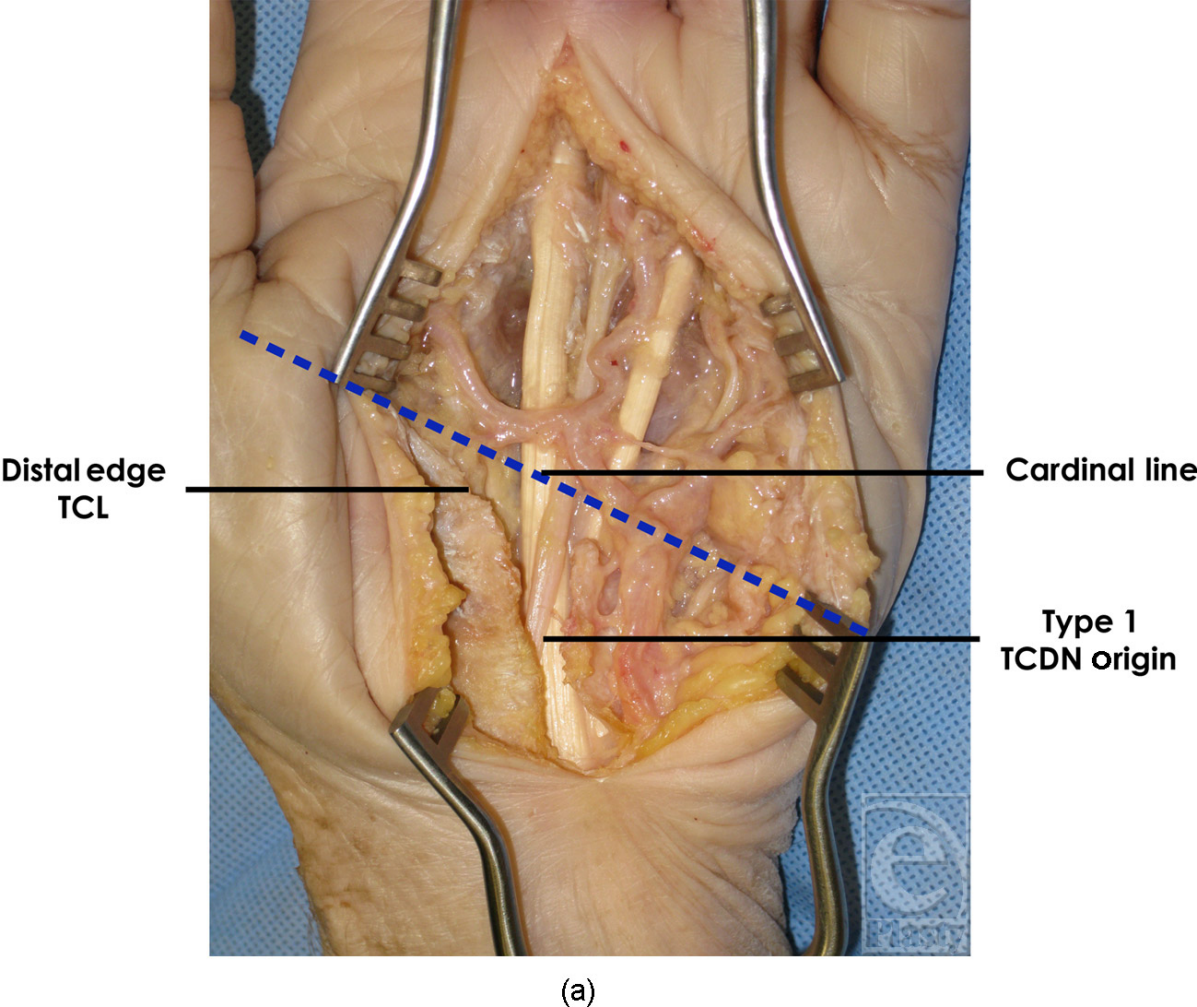
Origin of type 1 third common digital nerve (TCDN) origin. In 3 of 20 dissections, the TCDN originated from the median nerve within the carpal tunnel proximal to the distal edge of the transverse carpal ligament (TCL).

**Figure 5b:**
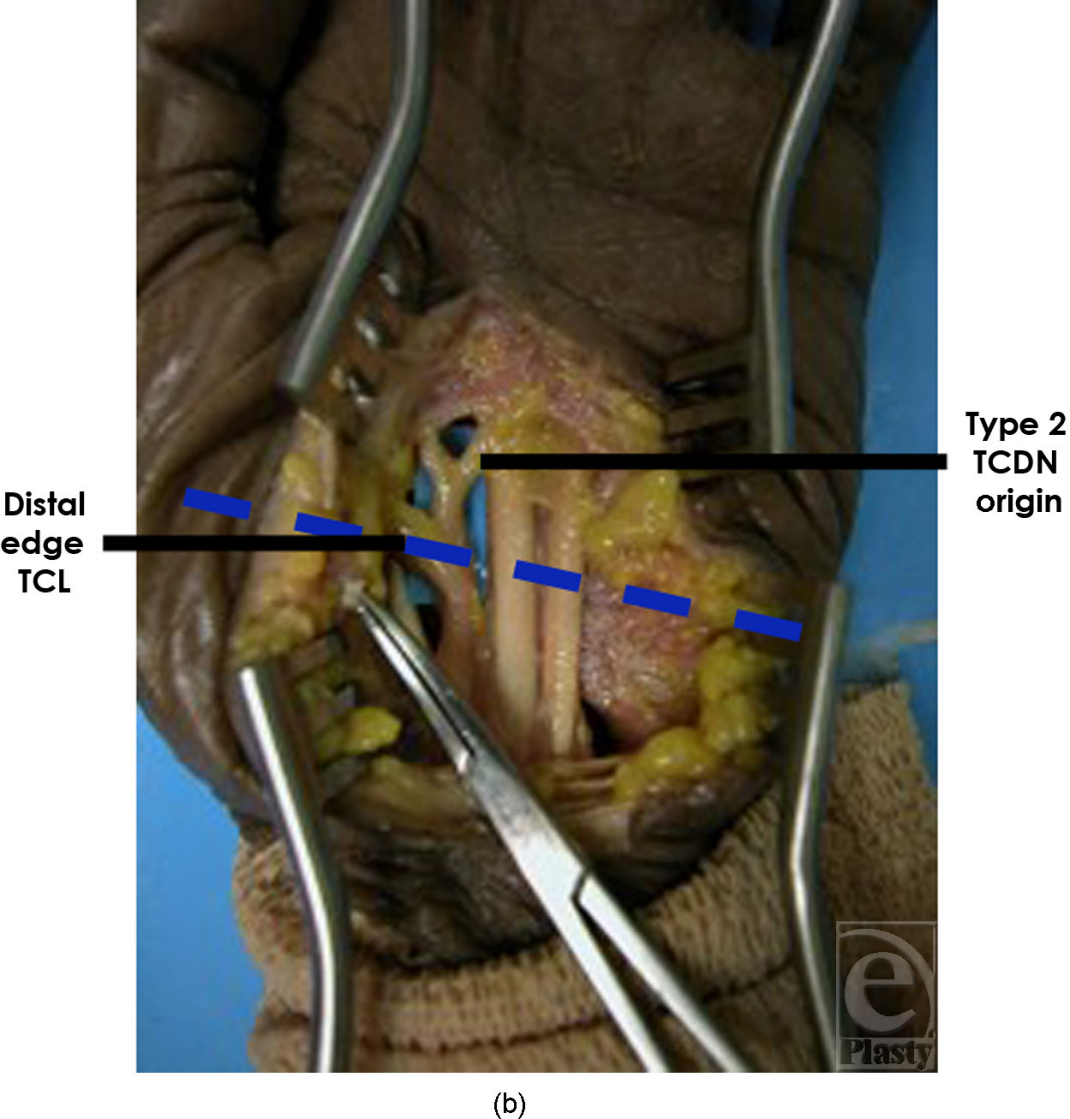
Origin of type 2 TCDN. In 14 of 20 dissections, the TCDN origin was found distal to the TCL but proximal to the superficial palmar arch.

**Figure 5c:**
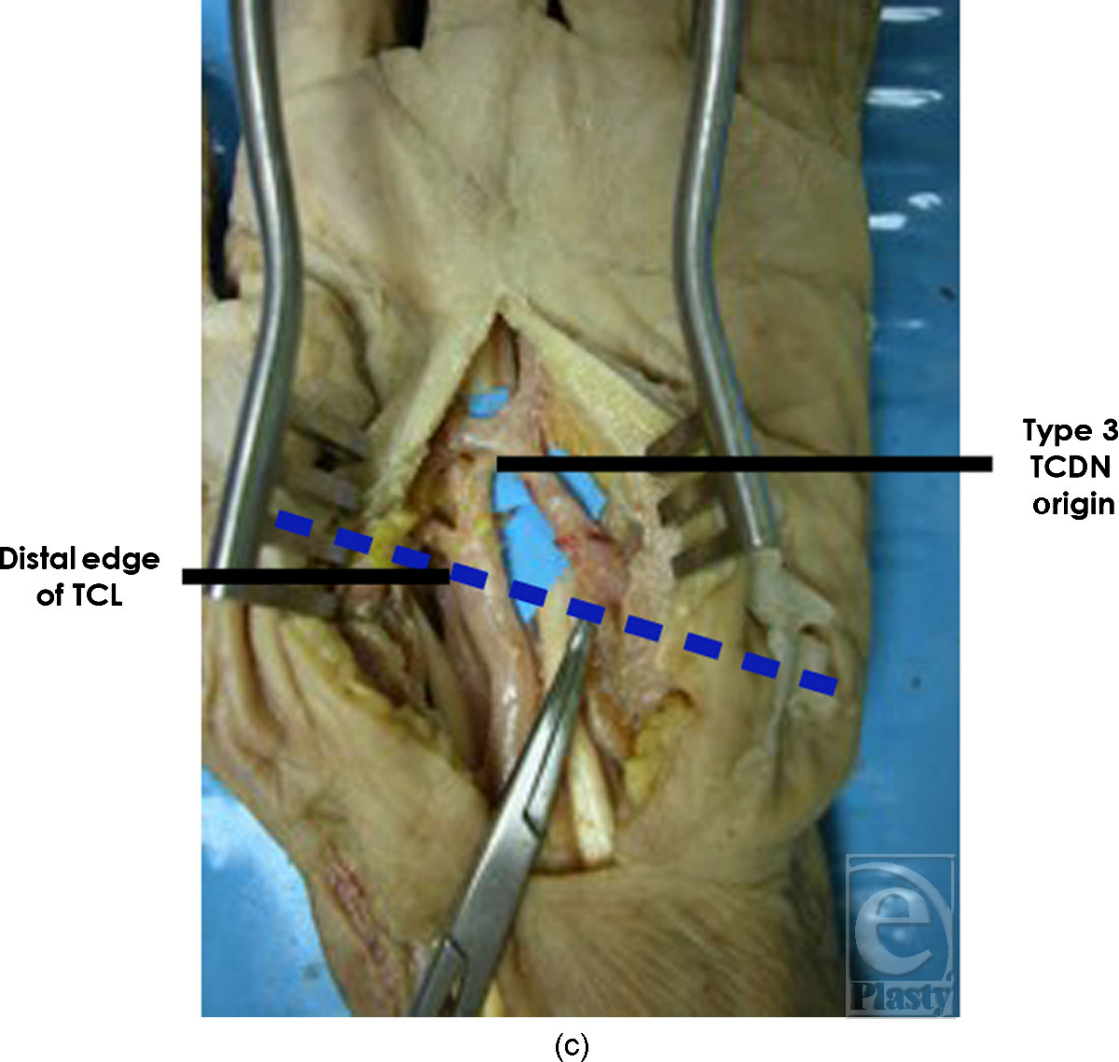
Origin of type 3 TCDN. In 3 of 20 dissections, the TCDN originated at or distal to the superficial palmar arch.

**Figure 6a:**
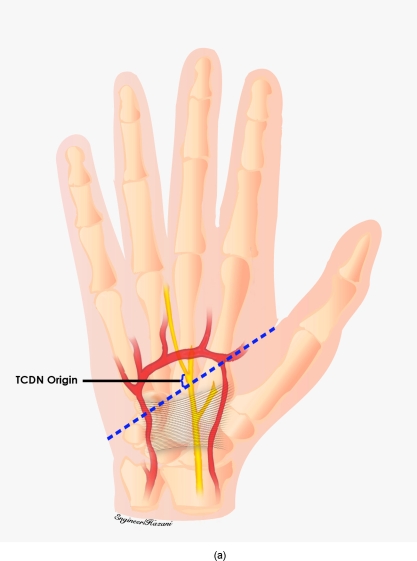
Third common digital nerve (TCDN) origin distance from cardinal line. On an average, the origin of the TCDN was measured at 5.0 ± 1.2 mm distal to the cardinal line (artist rendition).

**Figure 6b:**
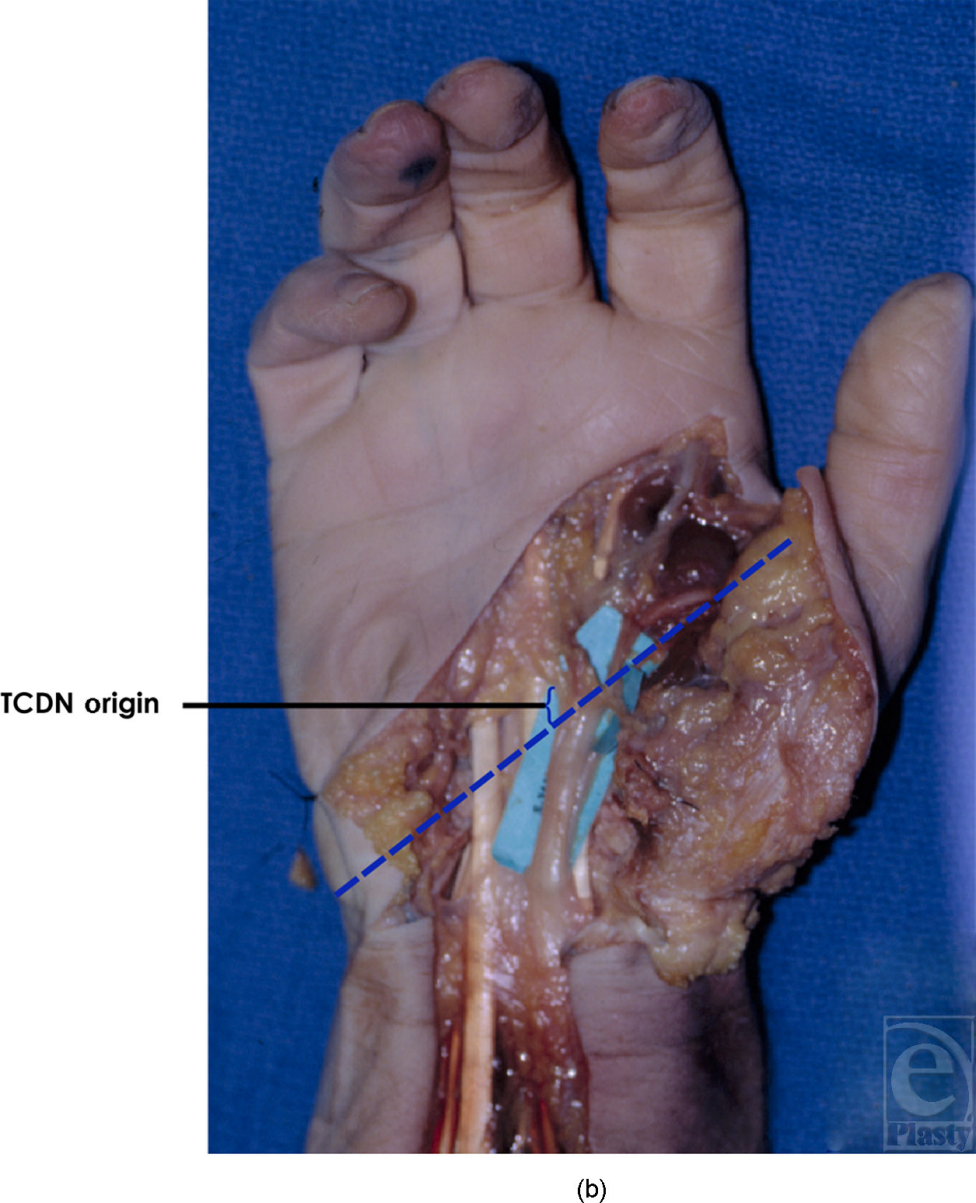
TCDN origin distance from cardinal line. On average, the origin of the TCDN was measured at 5.0 ± 1.2 mm distal to the cardinal line (cadaveric model).
